# Incompatibility of Antipyrin and Sweet Spirits of Nitre

**Published:** 1888-08

**Authors:** 


### INCOMPATIBILITY OF ANTIPYRIN AND SWEET SPIRITS OF NITRE.

Antipyrin, notwithstanding it is a patent medicine, (which fact is not generally known, however,) is being very extensively prescribed by physicians everywhere. The brilliant results which have been observed to follow its administration in some cases of fevers, have been enthusiastically reported in the medical press, and to-day "antipyrin" is the best advertised nostrum on the market.

It is a seductive drug ; the readiness with which it reduces the temperature in some cases ; its easy administration ; the freedom from the unpleasant effects which follow the administration of quinine, suggest it readily as a substitute for that drug ; and in it the doctors begun to believe they had found truly a *bonanza*. What more natural than the idea of combining it with other diaphoretics, and with diuretics to form an efficient febrifuge ? It would seem that a combination with sweet spirits of nitre, or with mindererus, or tincture of aconite, would furnish a very desirable "potion" for fevers.

But, to illustrate the great necessity of an acquaintance with the chemistry of all drugs prescribed in mixture, and the danger and folly of prescribing *secret* or *patent* medicines at all, it transpires that a mixture of antipyrin and sweet spirits of nitre developes a powerful poison,—and, we believe, more than one death has been caused by its administration. The subject has been discussed, lately, in several medical societies, in consequence of prominence given it by a certain newspaper report of death (or *alarming symptoms*) which followed the administration of a mixture of the kind in a certain Texas town, lately; and, actuated by a desire to enlighten our readers on the subject, as well as to warn them of the incompatibility and danger of the compound, Mr. O. Samostz, the practical chemist and pharmacist of Austin, at our request, instituted in his laboratory, a series of experiments with the following results :

By condensing phenyhydrocines with acet-acetic ether we receive methyloxychinicin $C6\ H5\ N2\ O3xC6\ H10\ O3{=}C10\ H10\ N2\ OxH1\ Ox\ C2\ H5\ OH$. If we then let iodide of methyl and caustic soda act on this methyloxychinicin we receive di-methyloxychinicin or antipyrin with the formula. — $C10\ Hg\ N2\ (C\ H3)\ O$.

Reaction of iodide of methyl and caustic soda on methyloxychinicin $C10\ H10\ N2\ OxNa\ O\ HxC\ H\ 3I{=}C10\ Hg\ N2\ (CH3)\ Ox\ H2\ Ox\ Na\ I$.

Tests have shown that after mixing an aqueous solution of antipyrin with an acidulous spirits of nitre (i. e. commercial spirits of nitre) the mixture assumes a green color, which will not appear, however, if the spirits of nitre has been neutralized or made slightly alkaline, say with ammonia, before mixing with the antipyrin solution.

Adding to the clear solution of spirits nitre and antipyrin a few drops of either nitric or muriatic acids, the green color reappears, thus showing that decomposition of the said alkaline mixture could be obtained by the acid of the stomach in case the antipyrin mixture was not alkalin enough to neutralize the acid of the stomach.

This same reaction occurs not only when mixing an antipyrin solution with acidulous spirits nitre, which represents the "nitrite of ethyl," but also when adding a solution of antipyrin with any other nitrite, for instance, nitrite amyl.

In this case, the same green color produced by the reaction, is obtained and proves to us that it is the nitrous oxide (representing the acid in the nitrite of ethyl, or amyl, as the case may be,) which causes the decomposition of the antipyrin in aciduous solutions, and forms the chemical substance with such toxic properties whose chemical composition may not yet be known ; as the formula of antipyrin is of such composition, in fact, the secrecy of its manufacture being entertained, thus allowing a great many different substances to result in its decomposition. It may be a new organic preparation with the effect of hydrocyanic acid, as it contains the same elements and series as the latter; however, the true composition may be determined by a more careful and concise qualitive and quantitive analysis. In conclusion, the continuous introduction of medicine of this class involves the necessity of a careful study of them, both by physicians and pharmacists, the aforesaid preparation being sometimes prescribed and sold at random by the druggists, like the old-time cathartic pill or quinine powder, regardless of the question, what are the effects of the antipyrin, antifebrin, which are both in their infancy when compared to the preparations whose place they should fill.

[The experiments were conducted by Edward Tips and Richard Schweickhardt, of Samostz' staff.—Ed.]

